# A study on different methods to change the Rayleigh number in the analysis of heat transfer

**DOI:** 10.1038/s41598-025-11120-9

**Published:** 2025-07-16

**Authors:** Vikrant Chandrakar, Atul Bhattad, Priyaranjan Samal, Jnana Ranjan Senapati, Abhishek Kumar Kashyap

**Affiliations:** 1 Department of Mechanical Engineering, Koneru Lakshmaiah Education Foundation, Vaddeswaram, 522502 India; 2 Department of Mechanical Engineering, National Institute of Technology, Rourkela, 769008 India; 3 Department of Mechatronics, Manipal Institute of Technology, Manipal Academy of Higher Education, Manipal, Karnataka 576104 India

**Keywords:** Natural convection, Rayleigh number, Characteristics length, Heat transfer, Gravity, Engineering, Physics

## Abstract

This study provides awareness about natural convection and associated non-dimensional numbers like the Prandtl number, Grashof number, Rayleigh number, and Reynolds number. The main focus of this research is to present the different methods employed to vary the Rayleigh number $\:\left(Ra\right)$ in an extensive range. The research concludes that changing the gravity value to obtain the considerable variation in $\:Ra$ is also a possible method for conducting the numerical analysis and observing the impact of the Rayleigh number. The validation of the numerical scheme with existing literature is provided here. An attempt is made to show that similar effects could be obtained by changing the value of gravity and the body’s characteristics length. The comparative results obtained by changing length and gravity are presented which gives almost the same result $\:(\pm\:\:10\%\:$error) to get the same $\:Ra$. This presents the beauty of a non-dimensional study. Moreover, it is possible to say that in the non-dimensional analysis of engineering practice, the individual variables that are changed are not important considering the non-dimensional results.

## Introduction

The exchange of thermal energy (heat) between physical systems is known as heat transfer. Conduction, convection, and radiation are the three ways that heat is transmitted. Conduction is the movement of heat through a substance as a result of energy exchange between molecules with higher and lower energies. Convection heat transfer is the type of heat transmission that happens as a result of the fluid’s bulk motion. The fluid moves in forced convection because of an outside force (such as a blower or fan), but in natural convection, temperature fluctuations in density cause the fluid to move. In radiation, the heat is transferred due to the motion of waves of energy (electromagnetic waves). All physical matters do radiate in the form of electromagnetic waves, and no medium is required for radiation.

In the present study, the prime interest is natural convection heat transfer. As the definition suggests, no external force or power is required for the fluid movement. So, the natural convection system has different advantages like minimum maintenance, cost is less, uncomplicated, and no noise generated due to the absence of moveable machinery. Due to these inherent advantages of natural convection systems, these are used in different plants and industries like mechanical, aeronautical, chemical engineering, manufacturing, cooling systems, and cryogenic plants. The flow in natural convection is buoyance-driven, making it far more interesting, unlike forced convection, where flow is relatively predictable. Few non-dimensional numbers are used in the natural convection study. The first one is the Grashof number $\:\left(Gr\right)$, and it is used in heat transfer and fluid mechanics. It is the ratio of the buoyancy force to the viscous force acting in the fluid layer. Its role in the free convection is as same as that of the Reynolds number $\:\left(Re\right)$ in forced convection. The $\:Gr$ characterizes the type of boundary layer developed in natural convection heat transfer. The $\:Re$ is the ratio of the inertia force and viscous force of the fluid, and it experiences relative internal movement due to various fluid velocities. It helps in characterizing the flow behavior (laminar or turbulent). Mathematically, the $\:Gr$ and $\:Re$ based on the characteristic length is expressed as^1,2^

1 $$Gr = \frac{{buoyant\:force}}{{viscous\:force}} = \frac{{g \cdot \beta \cdot \Delta T \cdot L^{3} }}{{\nu ^{2} }}$$

2 $$\text{Re} = \frac{{inertia\:force}}{{viscous\:force}} = \frac{{\rho \cdot v \cdot D}}{\mu }$$

In practice, both forced convection and natural convection occur simultaneously during heat transfer. However, the Richardson number (Ri)^3^ defined as $\:Gr/{Re}^{2}$ decides whether the flow is natural, mixed, or forced. Physically it is the ratio of the buoyancy term to the flow shear term.

3 $$\:Ri=\frac{Gr}{{Re}^{2}}=\left\{\begin{array}{c}\gg\:1;Natural\:convection\:flow\\\:=1;Mixed\:convection\:flow\:\\\:\ll\:1;Forced\:convection\:flow\end{array}\right.$$

Meanwhile, the Prandtl number $\:\left(Pr\right)$ is equally essential, which is a fluid property. It is the ratio of momentum diffusivity and thermal diffusivity. Mathematically represented as^4^

4 $$\:\text{Pr}=\:\frac{Molecular\:diffuscity\:of\:the\:momentum}{Molecular\:diffusivity\:of\:heat}=\frac{\nu\:}{\alpha\:}=\frac{\mu\:{C}_{p}}{k}$$

$\:Pr$ is the temperature-dependent property which means that every fluid has a specific Prandtl number at a particular temperature. Its values influence the relative growth of velocity and thermal boundary layer. If the $\:Pr$ is significantly less than 1 $\:(Pr\ll\:1),$ the thermal diffusivity dominates means heat disperses very quickly than the momentum, for example, in molten metal. Suppose the $\:Pr$ is very greater than 1 $\:(Pr\gg\:1),$ then the momentum diffusivity dominates, which signifies the very slow disbursement of the heat like in oils.

There is another number that is termed as Rayleigh number $\:\left(Ra\right),$ and it is majorly associated with buoyancy-driven flows, also termed natural/free convection flow. Rayleigh number is a product of $\:Gr$ and $\:Pr,$ and it means$\:\:Ra$ is a ratio of buoyancy to viscous force multiplied by the ratio of momentum diffusivity to thermal diffusivity. The main significance of $\:Ra$ is to decide whether the flow regime is laminar or turbulent. Also, $\:Ra$ is treated as an index for the determination of heat transfer characteristics in natural convection. Below the certain critical value of $\:Ra$, the fluid motion does not happen, and the mode of conduction only transfers the heat. Mathematically $\:Ra$ is expressed as^5,6^

5 $$Ra = Gr \cdot \Pr = \frac{{g \cdot \beta \cdot \Delta T \cdot L^{3} }}{{\nu \cdot \alpha }}$$

Generally, the flow regime is considered to be laminar when $\:Ra$ is less than $\:{10}^{8}$ while $\:Ra$ lies in between $\:{10}^{8}-{10}^{10}$ is treated as a transition regime and $\:Ra$ greater than $\:{10}^{10}$ signifies fully turbulent flow.

Natural convection within enclosures is a fundamental aspect of thermal-fluid research due to its relevance in numerous engineering applications, including passive cooling and energy systems. Recent computational studies have expanded the understanding of how various factors influence heat transfer and flow behavior in enclosed domains. Bezi et al.^7^examined how nanofluid properties affect entropy generation in a semi-annular cavity under unsteady convection, emphasizing the effect of enhanced thermal conductivity on reducing irreversibility. In related work, Souayeh et al.^8,9^ analyzed three-dimensional natural convection in cubical enclosures featuring internal heated cylinders and varying inclination angles, identifying critical conditions for the transition between steady and oscillatory flow regimes. Another study by Souayeh et al.^10^ assessed how different thermal conductivity ratios between solid obstructions and the surrounding fluid alter the internal flow patterns and thermal transport characteristics. Meanwhile, Hammami et al.^11^ conducted a numerical investigation of a two-sided lid-driven cavity, demonstrating how variations in lid velocity and cavity geometry can induce complex flow bifurcations. Several recent studies have focused on improving the understanding of heat transfer and fluid flow behavior in enclosures and ducts under various thermal and geometric conditions. Mathematical modeling of nanofluids has been explored to better predict thermal transport in such systems^12^. Mixed convection in ducts with moving walls and trapezoidal cavities containing discrete heat sources has been studied to highlight the impact of wall motion and cavity design on flow structure and heat transfer^13^. The stability of flow in vertical annuli with heat sources of varying lengths has also been examined, revealing how source length influences the onset of convection^14^. Natural convection between coaxial inclined cylinders was numerically investigated, showing how inclination affects temperature and flow distribution^15^. The role of fin size in magneto-convective heat transfer within wavy vertical enclosures has been studied using finite element analysis^16^. Hybrid nanofluids like MgO-SWCNT/water have been tested in zigzag cavities with internal obstacles to evaluate enhanced thermal performance^17^. Additionally, mixed convection in non-standard cavities connected to horizontal channels has shown complex flow behavior depending on the heat source location^18^.

The main focus of the present research that how $\:Ra$ can be varied in large ranges (like $\:{10}^{3}-{10}^{6}\:or\:{10}^{10}-{10}^{12}$) for numerical investigation. $\:Ra$ is a function of $\:g,\:\beta\:,\:\varDelta\:T,\:L,\nu\:,\alpha\:$ as per the definition. Any of these variables can be changed to get desired value of the Rayleigh number. In order to vary $\:Ra$ significantly (in the extensive range), few significant ways are available. The temperature difference $\:\left(\varDelta\:T\right)$, length of the object ($\:L$), changing the gravity value $\:\left(g\right),$ and viscosity of the fluid ($\:\nu\:$) can be varied significantly to get the changes in $\:Ra$ in the extensive range. There are few consequences observed while changing the $\:Ra$ using the mentioned method.

- The first method is changing the viscosity of the fluid ($\:\nu\:$). $\:Ra$ changed significantly using this method. Each fluid has a different viscosity with respect to temperature. It can change $\:Ra$ from a very low value to a very high value. But the disadvantage of this method is that every time one has to change the working fluid, which is not worthy of regressive investigation of any geometry. If the working fluid is fixed, the method fails.
- Another way is to change the temperature difference $\:\left(\varDelta\:T\right)$, which also causes a change in the thermal expansion coefficient ($\:\beta\:$). It is tough to change $\:Ra$ by this method. Because if one wishes to change the Ra, like 10^3^ from 10^5^ then they have to change the temperature in very high order, and the properties of many working fluids (ex., air) may not be available at such a higher temperature. So, this method is usually avoided.
- $\:Ra$ is also described as a product of $\:Gr$ and $\:Pr$. The $\:Ra$ can also be varied by changing the $\:Pr$ in the desired range. In the open literature, many authors^19–25^ obtained the desired $\:Ra$ by changing the $\:Pr$.
- $\:Ra$ can be changed significantly by changing the length $\:\left(L\right)$ of the object. If one wishes to go to a higher $\:Ra$, like 10^13^ from 10^7^, then the length has to be changed 100 times. When the length becomes 100 times, the geometry and grids are to be redrawn, and the computational domain has to have too many cells, and finally, a solution might be impossible. However, authors^26–34^ varied the $\:Ra$ by the length of the object for smaller range of $\:Ra$.

So, parametric studies with different Ra would not be possible, especially where different configurations or different geometries have been taken for the analysis. So, the best practical way of getting higher $\:Ra$ is to change the gravity, *g*, by some factor, which we are going to do in the present report. This gives the same result (non-dimensional form) if we change other parameters to get the same $\:Ra$ because that is the beauty of a non-dimensional study.

Since we are used to thinking *g* = 9.81 m/s^2^, any other value of *g* gives us a feeling that the problem is hypothetical. But it is not the case here. Since *g* occurs in $\:Ra$, we are studying the effect of Ra on other non-dimensional parameters, and not the effect of *g* is studied here. This is the beauty of non-dimensional analysis. Although it may appear that changing the value of *g* is hypothetical, in the non-dimensional analysis of engineering practice, the individual variables that are changed are not important considering the non-dimensional results. In fact, any other combination that gives the desired Ra would produce the same result. In order to justify the above statements, the present authors carried out a numerical study. The authors used different methods (changing the length of the object and the value of gravity) to change the $\:Ra$ and obtained the results. In the literature, many authors^35–42^ carried out their research for a large range of $\:Ra$ by changing the value of gravity. In order to justify exceptionality of the present work compared to the available literature, the key contributions of this study are as follows:

- Unlike many existing studies that emphasize flow visualization and contour-based interpretation, our study is centred on a quantitative validation of results using well-established benchmark data, emphasizing accuracy and reliability of the numerical approach.
- Here, a targeted analysis of how varying the Rayleigh number through adjustments in gravitational acceleration and characteristic length can be used as a controlled strategy to study natural convection. This perspective is often underexplored in conventional Rayleigh number studies.
- By not anchoring the results to a specific physical fluid or domain size, the study demonstrates a scalable and non-dimensional framework that can be applied across a broad range of thermal convection problems.

## Problem description, mathematical modelling and numerical procedure

Geometries like circular and cylindrical have long piqued interest in study because of their usefulness in electronic components, compressors, heat exchangers, gas turbines, and air conditioning systems. For many years, the focus of natural convection research has been on cavities and cylinders. The aim of this study is to investigate the thermo-fluid behavior surrounding a vertically oriented solid cylinder. The analysis involves solving the fundamental governing equations namely the continuity, momentum, and energy equations along with the $k - \epsilon$ turbulence model where applicable. These equations are discretized using the finite volume method, transforming the partial differential equations into algebraic forms. The resulting equations are then solved using the multi-grid solver available in ANSYS Fluent 15.0, with appropriate boundary conditions applied: a fixed wall temperature on the cylinder surface and ambient pressure and temperature conditions across the computational domain. To facilitate initial convergence, a first-order upwind scheme is applied to the convective terms in the momentum and energy equations. After achieving a preliminary solution, a switch to a second-order upwind scheme is made to improve solution accuracy. Diffusion terms are discretized using the central difference method. Pressure-velocity coupling is managed through the SIMPLE algorithm, and since the study includes buoyancy effects, pressure is discretized using the body force weighted scheme. The $k - \epsilon$ model is employed to simulate turbulence due to its reliability, robustness, and wide applicability, offering a good balance of stability and accuracy. Convergence criteria are set such that residuals must fall below 10^− 4^ for both continuity and momentum equations, and below 10^− 6^ for the energy equation.

**Fig. 1:**
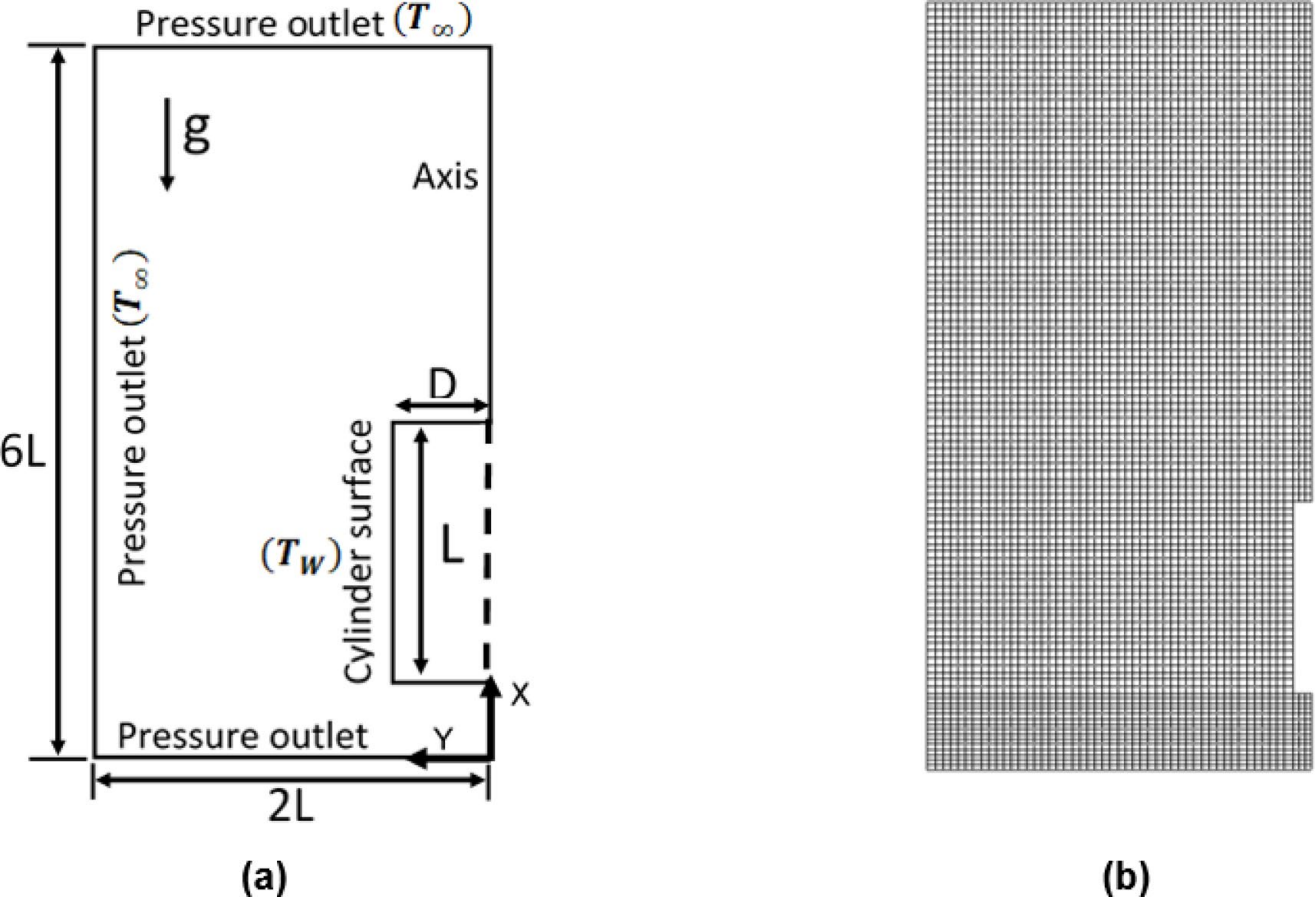
(**a**) axisymmetric geometry for the solid cylinder; (**b**) grid arrangement in the domain of computation.

The numerical investigation is carried out to obtain the Nusselt number at various $\:Ra$ from the simple geometry like a solid cylinder, and results are compared with existing correlations for the vertical solid cylinder. In order to avoid repetition of the governing equations, they are not mentioned here but those are available in already published work of the authors^43,44^. Figure 1 (a) illustrates an axisymmetric geometry of the vertical solid cylinder and the computational domain around it. Figure 1 (b) presents the distribution of grid around the cylinder. To change $\:Ra$, mainly two methods have been adopted for the present study, and those are by changing the length $\:\left(L\right)$ of the cylinder and by changing the acceleration due to gravity $\:\left(g\right)$. The temperature difference $\:(\varDelta\:T)$ between the ambient (300 K) and cylinder surface (350 K) is maintained at 50 K. The air is a working fluid that is non-newtonian, incompressible, single phase and steady in nature.

## Validation of the scheme and results

Before proceeding to the main simulation results, the authors conducted validation of the numerical scheme. Let us consider a flat vertical surface and calculate the Nusselt number $\:\left(Nu\right)$ for the case, and $\:Nu$ is the function of $\:Gr$. For natural convection problems, $\:Gr$ depends on the characteristics length, which is the height of the vertical surface. For a particular scenario where the thickness of the boundary layer (*BL*) and diameter of the cylinder (*D*) is of the same order or the *BL* thickness is less than *D*, then the same relationship could be used to calculate the heat transfer rate for both the vertical plate and vertical cylinder. Gebhart et al.^5^ provided the general criteria through which the vertical cylinder can be considered as a vertical flat plate, and it is given as

6 $$\:\frac{D}{L}\ge\:\frac{35}{{{Gr}_{L}}^{0.25}}$$

However, if the above criterion is not satisfied by any vertical cylinder, Minkowycz and Sparrow^45^ provided the multiplication factor *F* for gases from their analyses to include the curvature effect to consider the vertical cylinder as a vertical flat plate for $\:Pr$ of 0.7 and $\:F$ is mathematically given as,

7 $$F = 1.3 \cdot \left[ {\left( {L/D} \right)Gr_{d} } \right]^{{0.25}} + 1.0$$

The correlation for calculation of $\:Nu$ from the vertical cylinder subjected to natural convection developed by the Churchill^46^, Eq. (8), McAdam^47^, Eq. (9), and Eckert and Jackson^48^, Eq. (10) based on their experiments are written as follows for ready references.

8 $$Nu = F\left[ {0.825 + \frac{{0.387\;\left( {Ra} \right)^{{1/6}} }}{{\left[ {1 + \left( {\frac{{0.492}}{{\Pr }}} \right)^{{9/16}} } \right]^{{8/27}} }}} \right]^{2} \;10^{{ - 1}} \le Ra \le 10^{{12}}$$

9 $$Nu = 0.59F\left( {Ra} \right)^{{0.25}} \;10^{4} \le Ra \le 10^{9}$$

10 $$Nu = 0.021F\left( {Ra} \right)^{{0.4}} \;10^{9} \le Ra \le 10^{{13}}$$

**Fig. 2:**
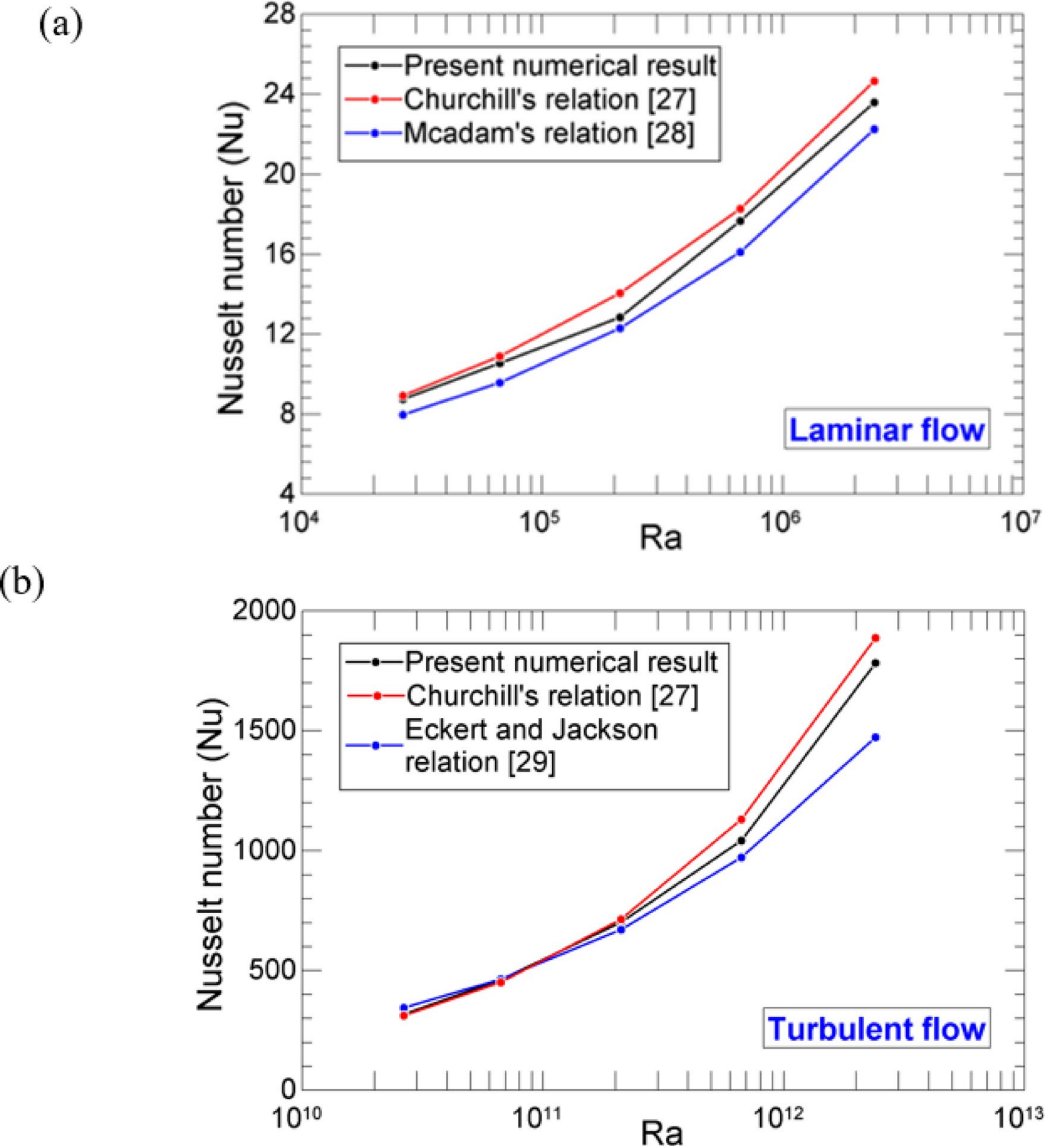
$\:Nu$ variation with $\:Ra$ to get the comparative results between the experimental correlation and present computational results.

Figure 2 shows the Nusselt number $\:\left(Nu\right)$ variation with Rayleigh number ($\:Ra$) for the vertical solid cylinder. This graph provides the comparative results from the present numerical analysis and experimental correlation. The Rayleigh number is varied by changing the gravity values$\:\:\left(g\right)$. Figure 2(a) shows the results for the laminar regime, while Fig. 2(b) shows the results for the turbulent regime. From Fig. 2, it is clear that results obtained from the present computational method follow a similar pattern as that of the experimental correlation, which shows an acceptable match between them.

**Table 1:** Nu values for different Rayleigh numbers at ∆T of 50 K.

$\:Nu$ $\:\varDelta\:T$			
Length $\:\left(\varvec{L}\right)$	$\:{\varvec{R}\varvec{a}}_{\varvec{L}}$	Gravity $\:\left(\varvec{g}\right)$	$\:{\varvec{N}\varvec{u}}_{\varvec{s}\varvec{i}\varvec{m}\varvec{u}\varvec{l}\varvec{a}\varvec{t}\varvec{i}\varvec{o}\varvec{n}}\left(\varvec{f}\varvec{o}\varvec{r}\:{\varvec{R}\varvec{a}}_{\varvec{L}}\right)$
0.02	$\:2.64\times\:{10}^{4}$	9.81	7.962
0.04	$\:2.11\times\:{10}^{5}$	9.81	12.86
0.09	$\:2.41\times\:{10}^{6}$	9.81	25.18
2	$\:2.64\times\:{10}^{10}$	9.81	320.7
4	$\:2.11\times\:{10}^{11}$	9.81	702.1
9	$\:2.41\times\:{10}^{12}$	9.81	1650

To justify the statements made by the authors that two primary methods are available to change $\:Ra$ in the extensive range for both laminar and turbulent flows are changing the length of the object $\:\left(L\right)$ or changing the value of gravity $\:\left(g\right)$. The significance has already been mentioned in previous paragraphs. Now, the authors have taken the six lengths for the solid cylinder to get the different $\:Ra$. Also, numerical simulation is carried out for a fixed length of the cylinder, and the gravity value is varied. It is found that the result matches within ± 10% of the error range. The results are shown in Table 1.

Table 2 shows the results obtained from the simulation. For the presented results, the authors created a solid cylinder of length $\:\left(L\right)$ 40 mm. After that, the different $\:Ra$ is obtained for fixed temperature change $\:(\varDelta\:T)$ equal to 50 K and mentioned length by varying the gravity term value $\:\left(g\right)$. $\:g$ value and its corresponding $\:Ra$ are mentioned in Table 2. Now, the obtained $\:Nu$ results are compared with $\:Nu$ results obtained from the $\:{Ra}_{L}$ by changing the length (as shown in Table 1) and $\:{Nu}_{g}$ by changing gravity. It is found that the error lies under 10%. Similarly, the authors fixed the length of the cylinder to 4 m and varied value to get the desired which is obtained from the changing length (as shown in Table 1). After that, the results are compared (as shown in Table 3) for obtained from changing and based on the. It is found that the error percentage lies within 8%.

**Table 2:** Comparison of the result obtained for laminar flow.

Length $\:\left(L\right)$	Gravity $\:\left(g\right)$	$\:{Ra}_{Length}$	$\:{Nu}_{simulation}\:\left({Nu}_{g}\right)$	$\:{Nu}_{{Ra}_{L}}$	Error
0.04	1.224	$\:2.64\times\:{10}^{4}$	8.761	7.962	0.100352
0.04	9.81	$\:2.11\times\:{10}^{5}$	12.86	12.86	0
0.04	111.8	$\:2.41\times\:{10}^{6}$	23.61	25.18	0.06235

**Table 3:** Comparison of the result obtained for turbulent flow.

Length $\:\left(L\right)$	Gravity $\:\left(g\right)$	$\:{Ra}_{Length}$	$\:{Nu}_{simulation}\:\left({Nu}_{g}\right)$	$\:{Nu}_{{Ra}_{L}}$	Error
4	1.224	$\:2.64\times\:{10}^{10}$	317.3	320.7	0.0106
4	9.81	$\:2.11\times\:{10}^{11}$	702.1	702.1	0
4	111.8	$\:2.41\times\:{10}^{12}$	1784	1650	0.081212

Some more insights can be drawn to understand the Rayleigh number importance in non-dimensional analysis as follows:

- As the Rayleigh number increases, either due to higher gravitational acceleration or a larger characteristic length, the buoyancy forces become stronger relative to viscous damping and thermal diffusion. This leads to more vigorous fluid motion, which enhances convective heat transfer in the domain.
- At lower Rayleigh numbers, the flow remains relatively stable and weak, often characterized by slow-moving thermal plumes and thicker boundary layers. However, as the Rayleigh number increases, the buoyancy-driven flow becomes more intense, resulting in stronger circulation patterns, thinner thermal boundary layers, and an overall increase in heat transfer rate.
- An increase in the characteristic length significantly amplifies the Rayleigh number (due to the cubic dependence on length), which directly influences the scale and strength of the convective cells. Larger domains allow for the development of more complex flow structures and can even promote the onset of unsteady or chaotic convection at sufficiently high Rayleigh numbers.
- Increasing gravity enhances the buoyancy forces driving the flow. Physically, this results in stronger upward movement of hot fluid and downward sinking of cold fluid, which leads to sharper thermal gradients and more efficient convective transport. This is especially evident in the central and boundary-layer regions where flow velocity and temperature gradients become steeper.

## Conclusion

The Rayleigh number is one of the most critical parameters in investigating natural convection heat transfer and buoyancy-driven flows. Different methods are discussed for changing $\:Ra,$ and it is found that changing the object’s length and gravity’s value is the most feasible option to obtain the variation in an extensive range (for high $\:Ra$ flows). But convenience wise changing length to get high $\:Ra$ is impractical in many cases. Like, if one wishes to go to a higher Ra, like 10^13^ from 10^7^, then the length has to be changed 100 times. When the length becomes 100 times, the geometry and grids are to be redrawn, and the computational domain has to have too many cells, and finally, a solution might be impossible due to computational constraints. So, parametric studies with different Ra would not be possible, especially where different configurations have been taken for the analysis. So, the best practical way of getting higher Ra is to change the gravity, *g*, by some factor. The comparative results obtained by changing $\:Ra$ and $\:g$ are presented in this report. This gives almost the same result $\:(\pm\:\:10\%\:$error) if we change other parameters ($\:like\:L\:and\:g$) to get the same Ra. This is the beauty of a non-dimensional study. In the open literature, there are few authors^35–38,41,43^ who have already used this method of changing the $\:g$ value to get high $\:Ra$ flows. They appropriately altered the gravity value to obtain the high $\:Ra$ (in the turbulent regime) for the maintained temperature difference and length of the object. Moreover, the Ansys-Fluent manual^49^ (Sect. 13.2.4.6.1, page no. 769 ) does suggest varying Ra by changing the gravity for solving high Rayleigh number flows. Moreover, the contours for temperature, pressure and velocity are not provided in the present research to make this research dedicated for the discussion of method in terms of quantitative way by validating the outcomes from the present methodology with the well-known existing literature. Also, similar studies had been already carried out by multiple authors in literature and presented their results in pictorial form of contours for various Rayleigh number using the above mentioned method.

The key contributions of this study which makes it different from the existing one is a quantitative validation of results using well-established benchmark data, emphasizing accuracy and reliability of the numerical approach. Unlike many existing studies that emphasize flow visualization and contour-based interpretation. Here, a targeted analysis of how varying the Rayleigh number through adjustments in gravitational acceleration and characteristic length can be used as a controlled strategy to study natural convection. This perspective is often underexplored in conventional Rayleigh number studies. Also by not fixing the results to a specific physical fluid or domain size, the study demonstrates a scalable and non-dimensional framework that can be applied across a broad range of thermal convection problems.

Therefore, based on the above discussion, it is possible to conclude that $\:g$ value can be changed for significant change (in order of tens or hundreds) in $\:Ra$ as the focus is to observe $\:Ra$ effect on the other non-dimensional parameters ($\:like\:Nu$). Although it may appear that changing the value of *g* is hypothetical, in the non-dimensional analysis of engineering practice, the individual variables that are changed are not important considering the non-dimensional results. The beauty of non-dimensional research lies in this. Although few researchers used the method of changing gravity for varying Ra in a broader range, a detailed report has not been done yet. To the best of the authors’ knowledge and belief, this research would be helpful for academic and research purposes. For the future work, authors will include varying additional parameters such as temperature difference and fluid properties, which will offer further insights and comparisons to provide a more comprehensive understanding of natural convection behavior.

## Data Availability

Data will be made available on request to first author or corresponding author.

## List of symbols

$$D$$: Cylinder diameter (m)
$$F$$: Multiplication factor
$g$: Acceleration due to gravity (m/s^2^)
$$Gr$$: Grashoff number
$$k$$: Thermal conductivity (W/m K^−1^)
$L$: Length of the cylinder(m)
$$Nu$$: Nusselt number
$$p$$: Pressure (N/m^2^)
$$Pr$$: Prandtl number
$$q$$: Heat flux (W/m^2^)
$$Q$$: Heat transfer (W)
$$Ra$$: Rayleigh number
$$Re$$: Reynolds number
$$Ri$$: Richardson number
$$T$$: Temperature (K)
${T}_{w}$: Cylinder surface temperature (K)
${T}_{\infty }$: Ambient air temperature (K)

## Greak letters

$$\alpha$$: Thermal diffusivity (m^2^/s)
$\beta$: Thermal expansion coefficient, 1/K
${C}_{p}$: Specific heat capacity at constant pressure J/kg K^−1^
$$\varepsilon$$: Emissivity
$\mu$: Dynamic viscosity of air (kg/m s^−1^)
$$\nu$$: Kinematic viscosity (m^2^/s)
$$\rho$$: Density (kg/m^3^)
$\sigma$: Stefan-Boltzmann’s constant (W/m^2^K^4^)

## Subscript

$g$: Based on gravity
$L$: Based on cylinder length
${\text{Length}}$: Based on length
${\text{Simulation}}$: Based on simulation
